# Love’s Stratagem*From “Recollections of a Rebel Surgeon.”

**Published:** 1899-10

**Authors:** 


### LOVE'S STRATAGEM.*

### The Doctor Puts Up a Job on the Major.

I always had a mighty sharp eye for pretty girls, said the Old Doctor, as he seated himself in our office chair. If there was one in the neighborhood, I'd find her. A regular "butter-fly-lover," I flitted from flower to flower, always deepest in love with the last girl I met.

There was one in the neighborhood when we were camped near Chattanooga, some two weeks before Bragg invaded Kentucky. I found her, of course, and "had it pretty bad." She lived down the valley some three miles below our camp. Her name was Mary Coffey. Her father was a rich, pompous old fellow named "General" Coffey. Why "General," I dont know; militia general once, I reckon, away back in the forties. In the South in those days, everybody who was anybody in particular had a military title, and the titles were graded according to one's importance in his vicinity, and ranged all the way from "Cap," bestowed on the postmaster and the city marshal, through "Major," the title of the editor; "Colonel," the title of the town lawyer and politician, to "General" for the fat, old rich fellows. Hence "General" Coffey, I suppose. He had the gout;—one foot all swelled up and bandaged, and he hobbled about, when he hobbled at all, on a stick and a crutch. He was a typical old-school gentleman of the South, hospitable, fond of company, a great talker and a great reader; had nothing else to do but talk and read, when he could get anybody to sit still and listen to him. His "overseer" attended to business,—the general was a planter,—and the general staid indoors mostly, taking his toddy, smoking his pipe and reading. He was a widower, and lived alone with his one child, this pretty daughter. Well, I became very fond of Miss Mary, and went to see her every night; but, confound it, the old general would hobble in the parlor and anchor himself and stay till I left. He had a yarn about some seven or eight foolish virgins who didn't keep their lamps trimmed, and got out of oil on a critical occasion (see the Bible). He drew an analogy between these negligent virgins and the Confederate government, applying it in some way, that I never did understand, altho' he told it to me every evening for a week. It

*From "Recollections of a Rebel Surgeon."

took him about an hour to tell it, and he would tell it with as much gusto and relish as if it were the first time. So, Mary and I could do nothing but grin and bear it, casting loving looks at each other whenever the old man would stoop over to spit; or "play-hands" on the sly. That would never do in this world. I'd get out of practice making shonuff love, and I was just dying to get Mary by herself. Love laughs at locks, they say. I set to work a scheme, and finally put up a job on the major. The major was a fat fellow named Robison, a bachelor, about forty years old. He was an aide, or something, on the general's staff; our general, not General Coffey. He was as vain as a peacock, a regular "masher," and prided himself on his (to him) good looks and his "conversational powers." Next day I said:

"Major, dont you want to call on a pretty young lady tonight?"

"Yes," said the major, as he glanced at himself in the little pewter-rimmed mirror hanging on the tent pole, and stroking his mustache lovingly, "who is she?"

"It's Miss Coffey, only daughter of General Coffey, a rich old Southern planter down the valley a little way. He's a fine old gentleman, a fine scholar, a great reader, and you will enjoy his society, I am sure, as only one of your literary attainments can," said I.

The major swelled with pride, and took another side glance at himself. "All right," said he; "we'll go tonight. The nights are lovely now; moon about full, and if there is anything I *do* love it is to talk to a pretty girl by moonlight."

I didn't say anything to this sentiment, tho' I could have said with Platt, "me, too," and added,—"yes, I see you at it now; something I have been trying to do for a week, but the general—" In stead, I said:

"Major, I ought to warn you now, that the general will talk you to death if you let him."

The major drew himself up proudly, and with a scornful look and a most conceited smirk, said:

"You forget, my son, that I'm a lady's man, and something of a talker, *myself*."

"All right," said I.

So, we went, that very night. The major got himself up in his best shape, dress parade uniform, epaulets, plumed hat and all; coat buttoned up to the chin, which must have been very uncomfortable, as it was yet September and pretty hot. He was so fat the buttons were on the strain, and he looked like a stuffed frog. I wore a

"fatigue" coat,—loose and easy-like. The major had a horse he called "Flop." I rode my little bay.

Entering the parlor on invitation of a servant, we found the general and Miss Mary both there.

"General Coffey,—this is my distinguished friend, the gallant Major Robison, of the general's staff; Miss Coffey, Major Robison."

After a cordial welcome, the general and the major were soon engaged in an animated running talk, the major getting in his licks with commendable and encouraging skill, and he was in fine spirits. I gave Miss Mary my arm, and excusing ourselves, we went out on the long front gallery in the moonlight. We staid out till eleven. Oh, it was a lovely night, indeed; full moon, cloudless sky, clear Southern atmosphere, and so still I could hear myself think what a good joke I was having on the major. The lovely valley, of which the gallery commanded a fine view, lay peacefully spread out before us, and there was nothing to suggest that "grim visaged war" was snoring all along the banks of the Tennessee, in about two miles of us, and that tomorrow we should see him shake himself and put on the Byronic "magnificently stern array." In fact, the stillness was unbroken, except by the barking of a little dog away over yonder, who, hearing the echo of his voice, would bark at it, and thus keep up the endless chain all night, I reckon. But I wasn't thinking of the night, nor the army, nor war, nor the valley, nor the little dog, just then. I was in better business. Yum, yum. Ever been there, Dan'els?

Byme-by Mary said:

"I reckon we'd better go in and see how father and the major are making it. It wont look right if we stay out all evening."

So, we went in. As we entered the light of the hall, she dexterously flipped off a little face powder, which had somehow gotten on the left breast of my coat, and picked off a long yellow hair, which somehow had gotten tangled on a button. We entered the parlor. The general had gotten the bulge on him and was doing all the talking, long since. The major, whose face was red, eyes ditto, jumped up quick, and swallowing a yawn, said:

"Well, doctor, it's about time we were going"; and was about to be off.

Miss Mary said: "Oh, it's early yet, and such a lovely night." (I could have hugged her, then and there, or anywhere else). I took out my watch. It was eleven o'clock. I didn't announce the fact, however, but said:

"Major, has the general told you his beautiful allegory of the seven virgins, yet?"

"No," said the old general, quickly; "I'm glad you reminded me of it. Sit down, major, and let me tell it to you."

And the major had to sit down, but he did it with a bad grace, and with a glance at me as dark as Erebus.

I again gave Miss Mary my arm, and asking them to excuse us, as we had had the pleasure of hearing it, we went out on the gallery again, and had another picnic. (More face powder and yellow hairs to brush off; yum, yum.)

I said it took the general an hour to tell the yarn. I knew just how to time our stay on the gallery, and made hay, figuratively, while the (moon) sun shone. Presently a rooster away over yonder waked up and gave the midnight signal. Another took it up and passed it down the line our way, till the general's chickens caught it, and repeated it about a thousand times, seemed to me; crowing for midnight. We went in. The general was nearing the climax, and was as wide awake as a mink. But the major. My stars! He was mad; mad as a wet hen. He was so mad he looked, as big as he was, to be actually swelled. His eyes were red; he was sleepy, sho-nuff, and couldn't swallow the yawns, but had to let them come out. He jumped up, cutting off the general about at "lastly," and was hardly civil in leave taking, notwithstanding the old gentleman's courteous invitation to call again, which was repeated so sweetly by Mary. He bolted out of the door and made for "Flop," muttering between his clenched teeth: "Yes, *I'll* call again." He was so mad he blowed like a porpoise; he even snorted. I didn't say a word; dasn't. Neither did he. We mounted in silence and rode away, I keeping just a little behind the major, and as mum as an oyster. We rode out of the lawn,—rode across the peaceful valley, up the slope of a hill, from the summit of which could be had a fine view of the old colonial manor house of the general's we had just left. Arrived at the summit the major turned his horse around, reined in; "Whoa, Flop," he said, and then, slowly and deliberately and for about a minit, shook his fist in the direction of the house, and said, with great deliberation:

"General Coffey; G—d——*d—n* you and your seven virgins and their oil!"

I fell off my horse and just rolled on the ground and hollered. I didn't go near the major for a week, and when I did he threw a rock at me.

